# A computer-aided diagnosis system for brain tumors based on artificial intelligence algorithms

**DOI:** 10.3389/fnins.2023.1120781

**Published:** 2023-07-07

**Authors:** Tao Chen, Lianting Hu, Quan Lu, Feng Xiao, Haibo Xu, Hongjun Li, Long Lu

**Affiliations:** ^1^School of Information Technology, Shangqiu Normal University, Shangqiu, China; ^2^Medical Big Data Center, Guangdong Provincial People's Hospital, Guangdong Academy of Medical Sciences, Guangzhou, China; ^3^Guangdong Cardiovascular Institute, Guangzhou, China; ^4^School of Information Management, Wuhan University, Wuhan, China; ^5^Department of Radiology, Zhongnan Hospital of Wuhan University, Wuhan, China; ^6^Department of Radiology, Beijing Youan Hospital, Capital Medical University, Beijing, China; ^7^Big Data Institute, Wuhan University, Wuhan, China; ^8^School of Public Health, Wuhan University, Wuhan, China; ^9^Institute of Pediatrics, Guangzhou Women and Children's Medical Center, Guangzhou Medical University, Guangzhou, China

**Keywords:** magnetic resonance imaging, computer-aided diagnosis (CAD) system, glioma, detection, grading, segmentation, classification, knowledge base

## Abstract

The choice of treatment and prognosis evaluation depend on the accurate early diagnosis of brain tumors. Many brain tumors go undiagnosed or are overlooked by clinicians as a result of the challenges associated with manually evaluating magnetic resonance imaging (MRI) images in clinical practice. In this study, we built a computer-aided diagnosis (CAD) system for glioma detection, grading, segmentation, and knowledge discovery based on artificial intelligence algorithms. Neuroimages are specifically represented using a type of visual feature known as the histogram of gradients (HOG). Then, through a two-level classification framework, the HOG features are employed to distinguish between healthy controls and patients, or between different glioma grades. This CAD system also offers tumor visualization using a semi-automatic segmentation tool for better patient management and treatment monitoring. Finally, a knowledge base is created to offer additional advice for the diagnosis of brain tumors. Based on our proposed two-level classification framework, we train models for glioma detection and grading, achieving area under curve (AUC) of 0.921 and 0.806, respectively. Different from other systems, we integrate these diagnostic tools with a web-based interface, which provides the flexibility for system deployment.

## Introduction

A brain tumor is a mass of tissue that is formed by an accumulation of abnormal brain cells. Most brain tumors are primary tumors that originate in the brain, and they are mainly benign without aggression to surrounding tissues (Neugut et al., 2019). The malignant brain tumors are cancerous and can spread to other part of the brain and central nervous system. Regardless of what type of brain tumor, early detection and diagnosis are crucial for appropriate therapeutic measures in order to improve the clinical outcomes and patients’ life quality (Yan et al., 2021). As one of the most common types of primary brain tumors, glioma can be categorized into different grades (from I to IV) by the degree of malignancy based on the World Health Organization (WHO) grading system (Louis et al., 2007, 2016). Among them, grade I is usually benign, and the remaining three grades are now usually categorized into the high-grade (WHO IV) and lower-grade (WHO II and III) (van den Bent, 2010; Reuss et al., 2015). Lower-grade gliomas have more favorable prognoses and longer survival times than high-grade gliomas. Also, distinct therapy strategies are used for these two subgroups of gliomas. Hence, accurate diagnosis and classification of gliomas are crucial for determining the best course of treatment and monitoring the progression of the disease.

As a non-invasive technique, magnetic resonance imaging (MRI) has been widely used in the clinical diagnosis of brain tumors by clinicians to characterize structural, cellular, metabolic, and functional properties of brain tumors (Villanueva-Meyer et al., 2017; Roberts et al., 2020). This technique can provide new insight into human brains for researchers. The related MRI types include structural MRI (sMRI), functional MRI (fMRI) and diffusion MRI (dMRI), which provide benefits of localized spatial information about the brain structure and function as well as structural and functional connectivity (Arbabshirani et al., 2017; Zhang et al., 2020). Among them, sMRI images of the brain usually deliver superior spatial and contrast resolution, thus suitable for studying various brain structures and for detecting structural abnormalities such as brain tumors (Arimura et al., 2009; Fangusaro, 2012; El-Dahshan et al., 2014). In recent years, the development of artificial intelligence, especially machine learning techniques, has promoted a lot of efforts using the sMRI modality to automatically detect gliomas (Kavin Kumar et al., 2018; Hussain et al., 2019; Kang et al., 2021), identify grades or subtypes of gliomas (Hsieh et al., 2017; Lu et al., 2018; Yang et al., 2018; Sajjad et al., 2019; Sengupta et al., 2019; Mitra et al., 2020; Zhuge et al., 2020; Tripathi and Bag, 2020, 2022a,b).

The results of the studies have, however, hardly ever been applied in clinical settings. The main cause of this is that, despite having claimed to get promising findings, the procedures suggested in many studies are complex and/or not intended to generalize to clinical data. Additionally, they either only give a single setup package that requires the difficult installation of numerous third-party data libraries and may depend on certain system architecture, or they do not offer clinicians user-friendly tools at all. In this study, we develop a novel computer-aided diagnosis (CAD) system for the detection and grading of gliomas. Models are first trained based on the features extracted from the available training data. The models are used by the CAD system to make a prediction on the test data. The CAD system is construed using a web-based architecture providing functions of preprocessing, prediction, segmentation, and knowledge-based guidance. The advantage of the web-based architecture is that we only need to deploy the machine learning tools and algorithms on a centralized server. The physicians can access the CAD system with a web browser installed on a standard PC. Additionally, it enables the continuous addition of new data to the training dataset to guarantee continual model performance improvement.

In a prior study, we developed a two-level histogram-based morphometry (HBM) classification framework by examining MRI images to identify autism (Chen et al., 2020) and glioma (Chen et al., 2021). In the first-level classification stage, the entire brain image was separated into a number of regions, and the histogram of gradients (HOG) (Dalal and Triggs, 2005) feature was extracted for each image region. Then a clustering method was used to transform each regional HOG feature into a high-level feature (e.g., diseased-related or healthy-related). Each region’s high-level feature was finally combined into a vector as a represent of the whole brain. In the second-level classification stage, these whole-brain vectors and labels were used to train a final classifier to make a prediction of the unknown data. When applied to the second edition of The Cancer Imaging Archive (TCIA) datasets and the Autism Brain Imaging Data Exchange (ABIDE) datasets, this methodology has shown encouraging results. Hence, in this glioma CAD study, we will also use the two-level HBM classification framework to the glioma diagnosis.

## Materials and methods

### Datasets

In this study, we trained two classification models based on two datasets. The first dataset is named as DS-Detect and used to identify whether the brain contains the glioma. In the DS-Detect dataset, the preoperative structural MRI (sMRI) data from June 2014 to December 2019 were collected from ZhongNan Hospital of Wuhan University. And this retrospective study on archived anonymized data was approved by the Ethics Committee of Zhongnan Hospital of Wuhan University. The second dataset is named as DS-Grade and used for glioma grading. In the DS-Grade dataset, the preoperative sMRI data were retrieved from TCIA database.^1^ The collection of original materials and data provided by TCIA was conducted in compliance with all applicable laws, regulations, and policies for the protection of human subjects.

The DS-Detect dataset contains 99 subjects including 62 patients with glioma and 37 healthy controls. Imaging was performed on a SIEMENS MAGNETOM Trio Tim 3.0 T MRI Scanner. Whole brain coverage was obtained with 23 contiguous 6 mm axial slices (TR = 7,000 ms, TE = 94 ms, TI = 2,210 ms, FA = 130, matrix size = 464 × 512). The DS-Grade dataset includes 134 subjects among which 76 are diagnosed as high-grade (WHO IV), and 58 as lower-grade (WHO II and III). Table 1 shows the subject characteristics in dataset DS-Detect and DS-Grade. Both datasets include three sMRI modalities: T1-weighted, T2-weighted, and T2-FLAIR. We chose T2-FLAIR modality since T2-FLAIR images are of higher-contrast and the high signal of tissue indicates the possible tumor growth. We also used three patients for blind test after training classification model based on these two datasets.

**Table 1:** Subject characteristics in the dataset for this study.

Characteristic	DS-detect		DS-grade	
	Patients	Healthy controls	High-grade	Lower-grade
Total number of subjects	62	37	76	58
Gender	Male: 41 (66.1%)	Male: 27 (73.0%)	Male: 51 (67.1%)	Male: 27 (46.6%)
	Female: 21 (33.9%)	Female: 10 (27.0%)	Female: 25 (32.9%)	Female: 31 (53.4%)
Mean age	62.5 ± 8.3 (41–77)	51.4 ± 10.2 (32–73)	57.6 ± 14.0 (17–81)	47.9 ± 14.1 (21–75)

### Data preprocessing

As the first step of image preprocessing, the MRIcron tool was used to convert the original DICOM scans of an individual into a single NifTI image file. Then we applied the bias correction and Z-score normalization methods, respectively, to address the issue of non-standardized MRI intensity values among intra-patient and inter-patient acquisitions. For the intra-patient intensity non-uniformity problem, we used the SPM12’s scan bias correction algorithm to minimize the inhomogeneity of MRI intensity within a tissue region. For the inter-patient intensity variability problem, we performed a *Z*-score normalization for each image, which normalize an image by simply subtracting the mean and dividing by the standard deviation of the whole brain, followed by clipping of the intensity value at [−4, 4] and a transformation to [0, 1]. Finally, we used the SPM12’s spatial normalization method to register all MRI images to the standard MNI space, which allows a meaningful comparison in a same place and at similar sizes.

### Overview of CAD system

**Figure 1:**
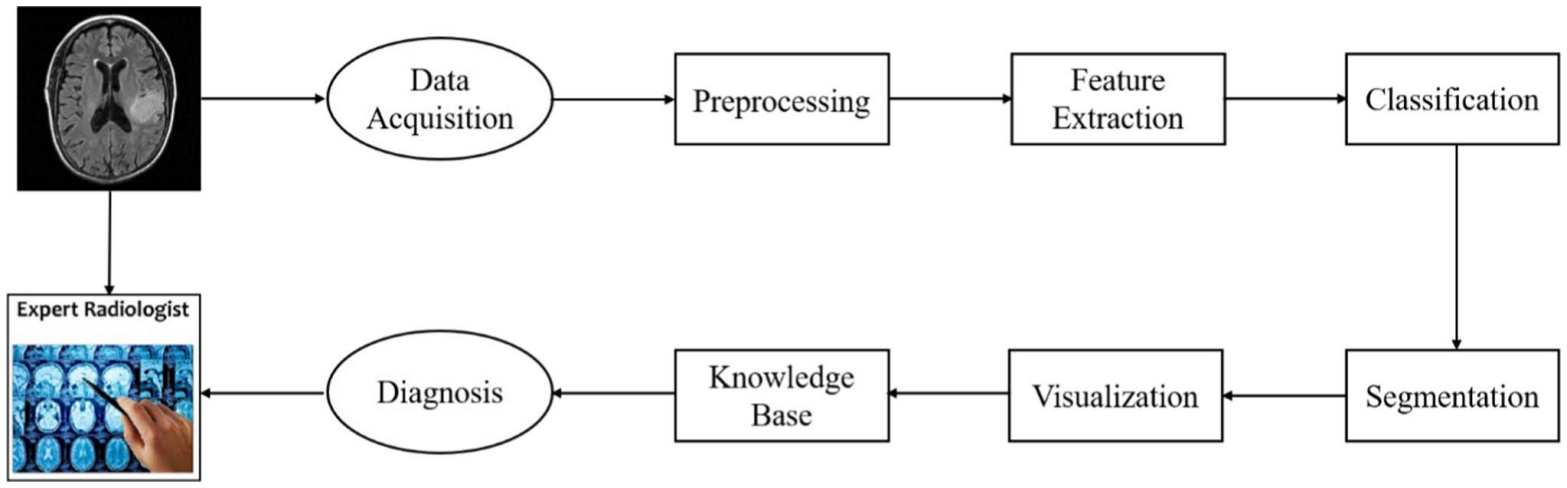
Diagnostic process for the glioma CAD system.

The goal of the CAD system presented in this paper is to detect gliomas and differentiate what grades the gliomas belong to. The architecture of the system is illustrated in Figure 1.

This figure shows the diagnostic process of an individual patient with glioma. First, the image data acquired from an MRI scanner are preprocessed using a standard pipeline. Then the regional HOG features are extracted from the preprocessed sMRI image. And these features are transformed into high-level features which are used to train classification models performing glioma detection or glioma grading. This CAD system also provides visualization of lesion boundaries for clinicians by using a semi-automatic segmentation method. Finally, the clinicians can acquire more diagnosis guidance from a brain tumor knowledge base.

**Figure 2:**
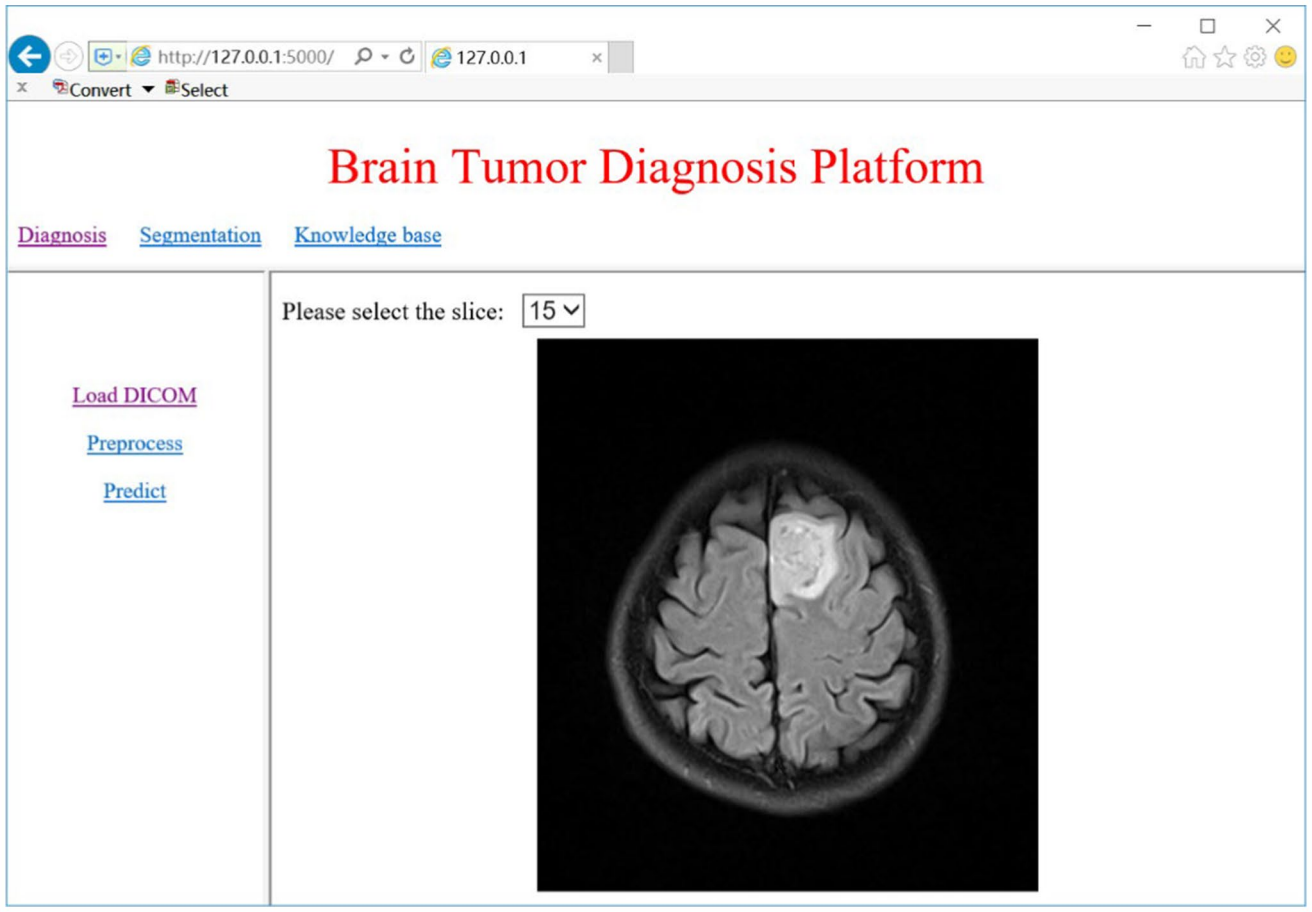
Web interface of the glioma CAD system for clinicians.

The CAD system can be divided into four modules: model training, glioma diagnosis (detection/grading), segmentation, knowledge discovery. The web interface of the system for clinicians is illustrated in Figure 2. In addition to the diagnostic process, we also provide the interface of model training for system administrator.

### Detection and grading

**Figure 3:**
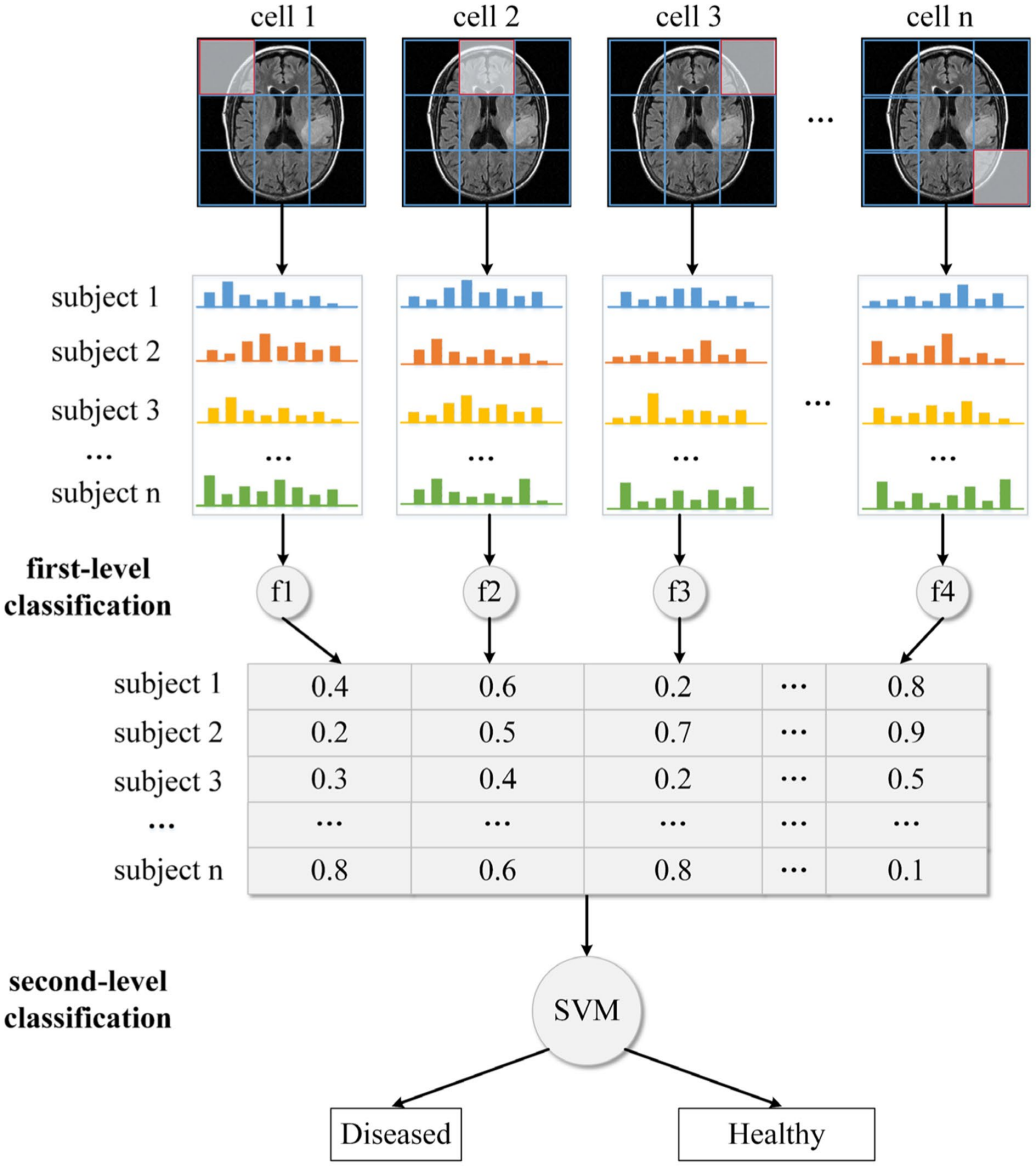
The two-level classification framework for glioma detection.

In this study, we use our recently developed two-level HBM classification framework to perform glioma detection or grading. Based on the DS-Detect and DS-Grading datasets, we train two machine learning models to perform different classification tasks. For the sake of convenient illustration, we take glioma detection as an example and only show one MRI slice in Figure 3. Actually, we use all the slices in the execution of the algorithm.

We divide the whole brain into various local regions/cells, as shown in Figure 3. Following that, each cell’s local HOG feature is extracted. In the traditional HOG application, these local features are combined into a large descriptor representing the entire image. Although the combined HOG descriptor can depict the MRI image in detail covering all pixel gradients, the high dimensionality of the feature vector and disease-unrelated information may lead to a reduction in model performance. Thus, instead of directly concatenating these local HOG features, we transform them into individual high-level features. Specifically, for all images in the training dataset, we apply the fuzzy c-means clustering method on HOG features with the same cell position. The centroids for the disease-related cluster and the health-related cluster are then obtained, together with fuzzy numbers that indicate the degree to which the features are disease- (or health-) related. Then all the transformed high-level features (e.g., fuzzy numbers) are concatenated into a feature vector which is used as input to train a final or second-level classifier based on the SVM method. And this final classifier is used to predict whether an unknown subject is healthy or diseased.

### Segmentation

The segmentation of glioma in MRI images allows quantitative analysis of clinical parameters related to volume and shape (Litjens et al., 2017). The task of segmentation is the assignment of each voxel in the MRI image to a specific category, based on the environmental information around the voxel. According to the summative work of other researchers (Anwar et al., 2018; Chen and Pan, 2018; Guo et al., 2018; Li et al., 2018), the main segmentation methods can be divided into two categories: region-based segmentation, and FCNN-based segmentation. Classifiers or feature extractors must be trained with labeled MRI images for both types of segmentation approaches. If the training MRI images are obtained from various scanners, there may be significant differences between them. Also, there are variations between the new MRI scans and the training MRI images. Despite the model performing well on the training dataset, these variations can result in poor model performance on the new MRI images. To eliminate those differences between MRI images, we proposed a novel semi-automatic segmentation algorithm named as Expanding Segmentation (*Exp-seg*) for the CAD system to identify the contour of glioma region slice by slice. The clinician should label some glioma voxels and some normal voxels in the representative slice containing the most gliomas before automatically executing Exp-seg. The convex polygons connected by the glioma voxels are defined as the initial glioma region, and the initial normal region is defined similarly.

### Algorithm framework

**Figure 4:**
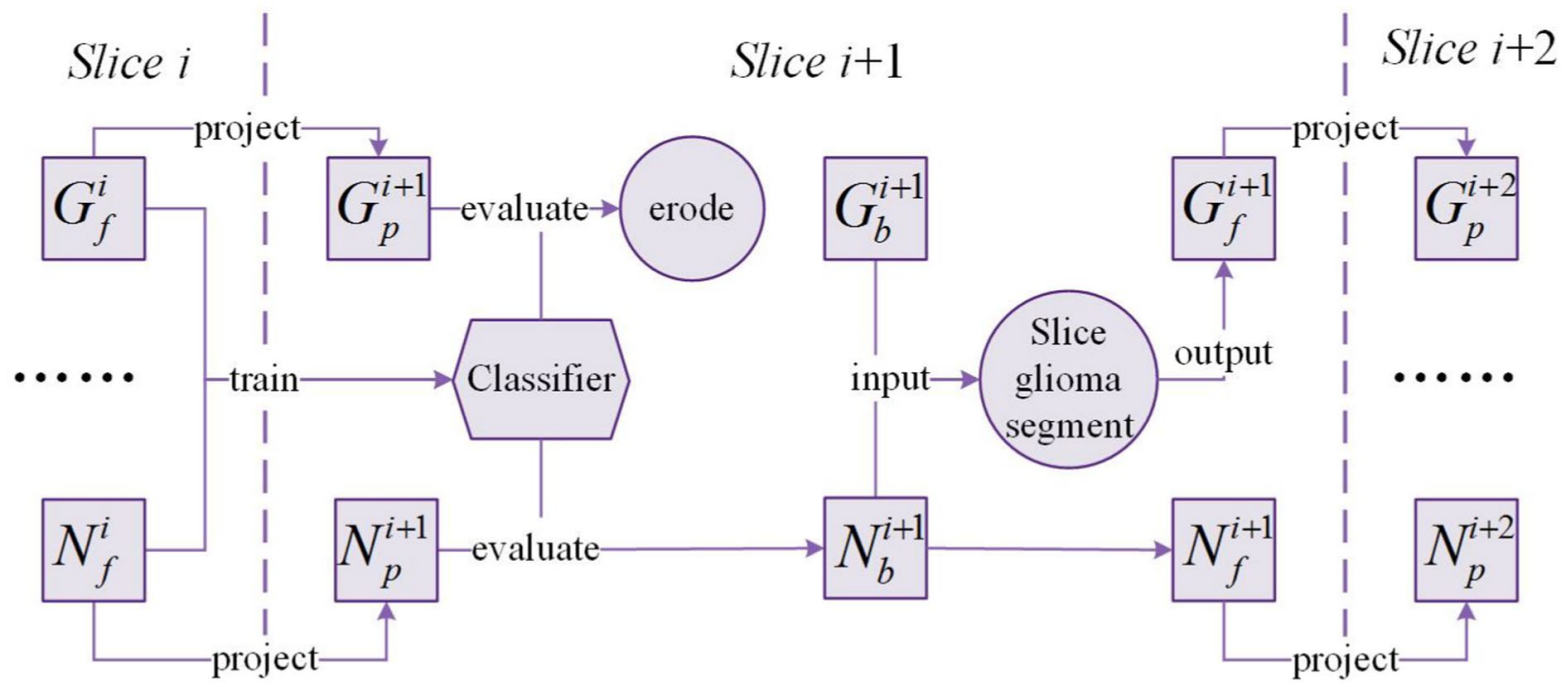
*Exp-seg* framework.

The framework of *Exp-seg* is shown in Figure 4. G and N denote the glioma region and the normal region in the brain MRI image, respectively. $G_{p}^{i}$ , $G_{b}^{i}$ , and $G_{f}^{i}$ denote the possible glioma region, the initial glioma region, and the final glioma region in the slice i respectively. Similarly, $N_{p}^{i}$ , $N_{b}^{i}$ , and $N_{f}^{i}$ denote the possible normal region, the initial normal region, and the final normal region in the slice i respectively.

After segmentation of slice i is finished, $G_{f}^{i}$ and $N_{f}^{i}$ are obtained. Then the masks of $G_{f}^{i}$ and $N_{f}^{i}$ are projected onto the adjacent slice i+1 where the brain region covered by the projected mask are defined as $G_{p}^{i+1}$ and $N_{p}^{i+1}$ respectively. Due to the differences between adjacent slices, we need to train a classifier based on the voxels in $G_{f}^{i}$ and $N_{f}^{i}$ to identify glioma and normal voxels in $G_{p}^{i+1}$ and $N_{p}^{i+1}$ . After evaluation by the classifier, the voxels classified as non-glioma in $G_{p}^{i+1}$ are removed, and the voxels classified as non-normal in $N_{p}^{i+1}$ are also removed. To minimize the number of misclassified voxels in $G_{p}^{i+1}$ , morphology erosion is performed after the evaluation.

In slice i+1 , $G_{b}^{i+1}$ is considered as the origin of the glioma region. Glioma segmentation is applied on $G_{b}^{i+1}$ by multiple times of expansion-segmentation that is named as slice glioma segmentation. Slice glioma segmentation can produce the final segmentation result in slice i+1 : $G_{f}^{i+1}$ and $N_{f}^{i+1}$ . The above process will be executed sequentially on the following slices until all glioma slices are segmented.

### Slice glioma segmentation

**Figure 5:**
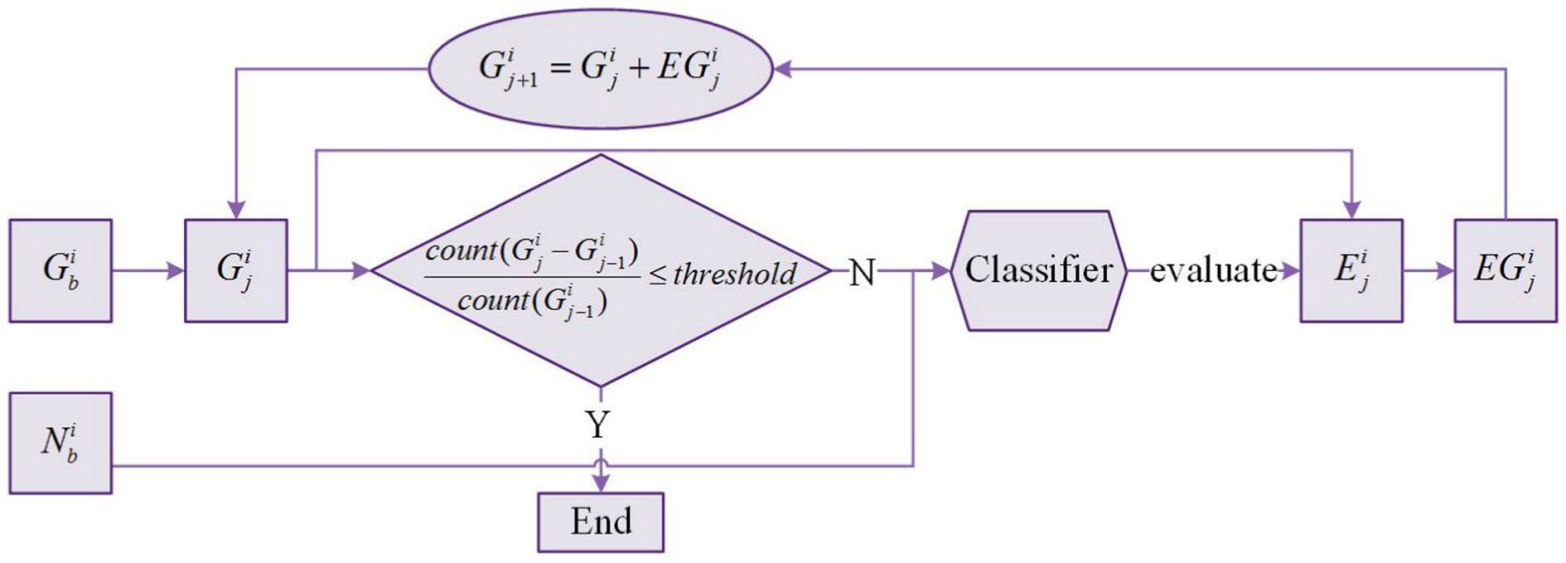
Slice glioma segmentation process.

Figure 5 shows the slice glioma segmentation process taking slice i for example. $G_{j}^{i}\left(j=1,2,\cdots \right)$ denotes the glioma region of jth segmentation in slice i , and $G_{1}^{i}=G_{b}^{i}$ . $E_{j}^{i}\left(j=1,2,\cdots \right)$ denotes the expanded region of jth segmentation in slice i . $EG_{j}^{i}\left(j=1,2,\cdots \right)$ denotes the expanded glioma region of jth segmentation in slice i . With $G_{b}^{i}$ and $N_{b}^{i}$ given, $G_{f}^{i}$ and $N_{f}^{i}$ can be obtained by slice glioma segmentation. The segmentation process is iterated multiple times until the stop condition is met.

Because the expanded region may include both glioma voxels and normal voxels. Thus, we need to train a classifier based on the voxels in $G_{j}^{i}$ and $N_{b}^{i}$ to recognize glioma and normal voxels in the expanded region. After iterating multiple times, almost all voxels in the expanded region are normal voxels. Therefore, the formula in Figure 5 is used as the iteration stop condition where count(x) is the function to count the number of voxels in x , and threshold is a hyperparameter ranging from 0 to 0.1. In this process, $G_{j}^{i}$ updates many times, and the last $G_{j}^{i}$ is the final glioma region $G_{f}^{i}$ . There is no change in $N_{b}^{i}$ , and $N_{b}^{i}$ is equal to $N_{f}^{i}$ .

### Knowledge base of brain tumors

**Figure 6:**
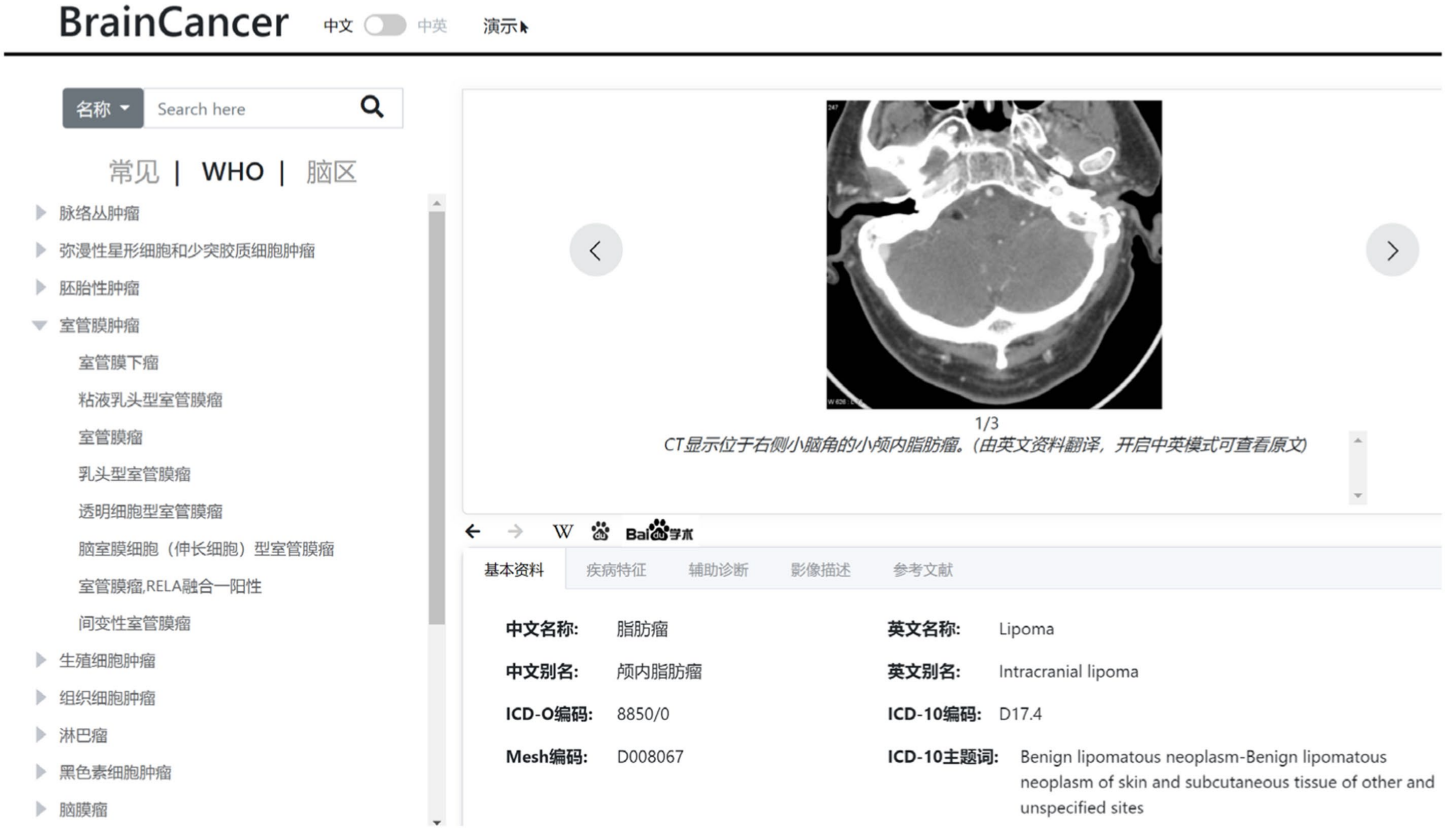
Web-based knowledge base of brain tumors.

In this study, we also develop a web-based knowledge base on typical brain tumors, which include data such as tumor profile, tumor characteristics, imaging description, auxiliary diagnosis, and reference literatures. This knowledge base is constructed according to the WHO classification system for tumors of the central nervous system. The typical brain tumors are listed on the left panel of Figure 6 in a tree-like layout. When a specific tumor type is selected or located *via* a search, the detailed information about the tumor will be displayed on the right panel of Figure 6, which can provide some guidance for the diagnosis of gliomas.

### Text mining

From descriptions of brain tumors, important data is extracted using text mining algorithms, such as tumor-prone brain areas, clinical manifestations, morbidity, and susceptible populations. The extraction of tumor-prone brain regions only involves named entities, while the extraction of morbidity and susceptible populations involves both entity and relationship. Before extracting information, we need to use open source tools like NLTK, Stanford NLP, and the Python jieba library for word segmentation and part-of-speech tagging. The extraction methods are mainly divided into two types based on rules or statistics. The WHO tumor classification system has a maximum of 200 tumors, whereas the statistical-based technique requires a large amount of training data. Hence, target information is extracted manually using a rule-based technique in this knowledge base.

### Ontology design

**Figure 7:**
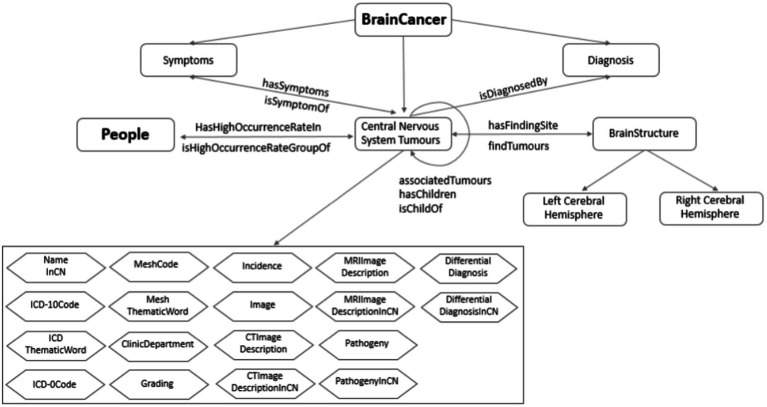
Ontology relationship network of knowledge base.

The ontology of brain tumor is constructed based on the tumor data including tumor profile, tumor characteristics, imaging description, auxiliary diagnosis, and reference literatures. Through the brain area and the susceptible population, respectively, the tumor ontology is related to the brain ontology and population ontology. The knowledge organization of the brain ontology is mainly composed of two dimensions: brain structure and blood supply system, while the population ontology is composed of susceptible population. On this basis, the ontology network of the resulting knowledge base is established as shown in Figure 7.

The main steps of the ontology relationship design are as follows:

First, according to the established brain tumor knowledge organization dimension, we obtain the semantic relationship related to brain tumors, including diagnosis, symptoms, susceptible population, susceptible brain area, and upper position tumor. Secondly, according to the functional requirements of the knowledge base, some inverse object attributes are inferred, including the disease symptoms, population susceptible, brain susceptible, and lower tumor. Finally, association tumors are established through text mining. There are three sources of similar diseases: literature, similar predictions, and reasoning. The similarity total score of tumors is calculated with the characteristics of three areas: tumor-prone brain regions, clinical manifestations and imaging findings. And the calculation includes the following steps: calculating feature score, preprocessing feature data, training feature weight, testing similar threshold, and predicting similar tumor.

We build an ontology model in Protégé, and fill in more than 170 types of tumor data collected. The amount of data is large, so we developed python programs to automatically fill them. We use OwlReady’s Python third-party library for ontology reading, editing, and saving.

## Results

One of the capabilities of the CAD system is to make a prediction of whether the brain scan images contain gliomas and what grade the gliomas belong to. The prediction accuracy depends on the performance of the training model. In this section, we first give an evaluation of the classifier trained on the DS-Detect and DS-Grading dataset, respectively. And then we provide a visualization of the glioma segmentation result using our proposed *Exp-seg* algorithm.

### Model performance evaluation

**Figure 8:**
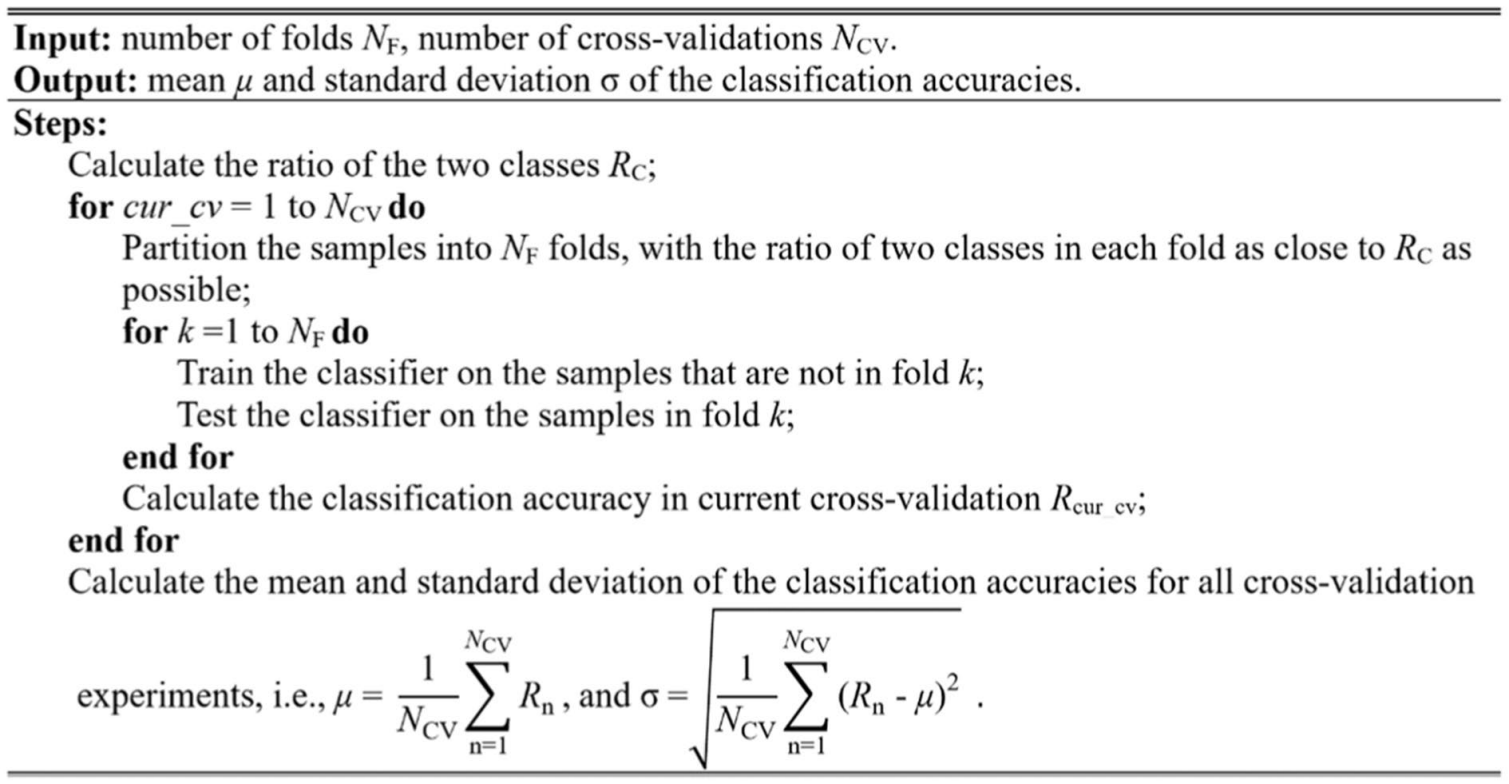
Algorithm of the stratified cross-validation with multiple random runs.

Cross-validation is typically used to evaluate the model performance. The widely used methods in brain image analysis is k-fold cross-validation, especially 10-fold cross-validation. In this study, we used the stratified 10-fold cross-validation method to evaluate the model performance. The stratified method ensures that the sample percentage for each of the classes in every fold is equal to that in all samples, retaining the original data distribution pattern of the entire dataset. Furthermore, the variance of the model will decrease by performing several random runs, each of which first shuffles the dataset and then splits it into a pair of training and test sets. The stratified cross-validation method proposed in this study is implemented as the pseudo-code shown in Figure 8.

In this study, we evaluated the model performance using the measurements such as accuracy (ACC), sensitivity (SEN), specificity (SPE), and area under curve (AUC). These measurements can be calculated from the classification confusion matrix. Here, the accuracy is defined as the ratio of correctly classified subjects over all subjects. The sensitivity is the ratio of correctly classified subjects with glioma over all subjects with glioma, and the specificity is the ratio of correctly classified subjects without glioma over all subjects without glioma. The AUC refers the area under the receiver operating characteristic (ROC) curve. The larger AUC value means better model performance. As mentioned above, cell size is a parameter that will affect the performance of the model. In the experiment, we assign the cell size with value from 10 to 20. Table 2 shows the cross-validation performance of the two-level classification framework.

The measurements from Table 2 are calculated after 10 random runs of stratified 10-fold cross-validation for each cell size. In glioma detection task, the model achieves the best performance when cell size equals 20 as the Table 2 shows. In glioma grading task, the model achieves the best performance when cell size equals 18. And the model performance of glioma detection is generally better than that of glioma grading. The reason why glioma grading is more challenging is that it needs to distinguish the subtle structural differences between different grades of gliomas.

**Table 2:** Cross-validation performance of two glioma classification tasks.

Cell Size	Glioma detection				Glioma grading			
	ACC (SD)	SEN (SD)	SPE (SD)	AUC (SD)	ACC (SD)	SEN (SD)	SPE (SD)	AUC (SD)
10	74.9% (2.8)	92.1% (2.1)	51.6% (4.9)	0.832 (0.012)	71.2% (1.8)	81.6% (1.5)	58.0% (3.2)	0.781 (0.017)
11	73.4% (1.5)	85.3% (2.6)	52.7% (3.7)	0.832 (0.015)	71.0% (2.6)	80.6% (2.9)	58.9% (2.7)	0.697 (0.013)
12	80.4% (2.8)	88.6% (2.4)	66.0% (5.5)	0.891 (0.011)	66.8% (1.7)	74.1% (2.7)	57.1% (2.2)	0.718 (0.024)
13	72.4% (1.3)	82.2% (1.3)	55.2% (4.1)	0.791 (0.017)	70.7% (1.3)	80.6% (2.6)	57.9% (3.2)	0.741 (0.015)
14	72.5% (2.5)	79.6% (2.3)	59.8% (5.1)	0.821 (0.021)	74.8% (2.4)	82.9% (3.9)	64.1% (1.9)	0.776 (0.015)
15	73.0% (2.7)	81.1% (2.6)	58.6% (6.1)	0.838 (0.019)	74.3% (2.9)	79.7% (2.6)	67.6% (4.8)	0.771 (0.022)
16	82.8% (1.9)	87.8% (2.6)	73.9% (4.3)	0.898 (0.015)	68.2% (2.0)	75.2% (3.3)	58.7% (2.7)	0.717 (0.021)
17	76.4% (2.4)	81.8% (3.2)	66.5% (4.8)	0.849 (0.017)	68.2% (2.1)	74.9% (2.1)	59.4% (3.1)	0.724 (0.023)
18	72.3% (2.5)	80.2% (2.4)	58.3% (3.8)	0.791 (0.015)	**76.3% (2.4)**	**83.7% (3.2)**	**68.7% (2.7)**	**0.806 (0.022)**
19	79.5% (1.8)	83.9% (1.8)	71.7% (4.4)	0.861 (0.014)	73.5% (3.5)	79.6% (3.6)	65.7% (4.7)	0.769 (0.017)
20	**86.3% (1.4)**	**89.4% (1.9)**	**80.5% (2.5)**	**0.921 (0.007)**	65.8% (2.6)	70.9% (2.2)	59.2% (3.8)	0.66 (0.022)

The bold values Table 2 are means the best performance of two classification tasks.

### CAD system evaluation

The CAD system can provide a pipeline for glioma diagnosis by analyzing the sMRI images including image preprocessing, glioma detection and grading, glioma segmentation, and related knowledge discovery. As Figure 2 shows, the CAD system provides web access for clinicians. Here we choose brain MRI scans from two subjects to evaluate the CAD system.

### DICOM preprocessing

**Figure 9:**
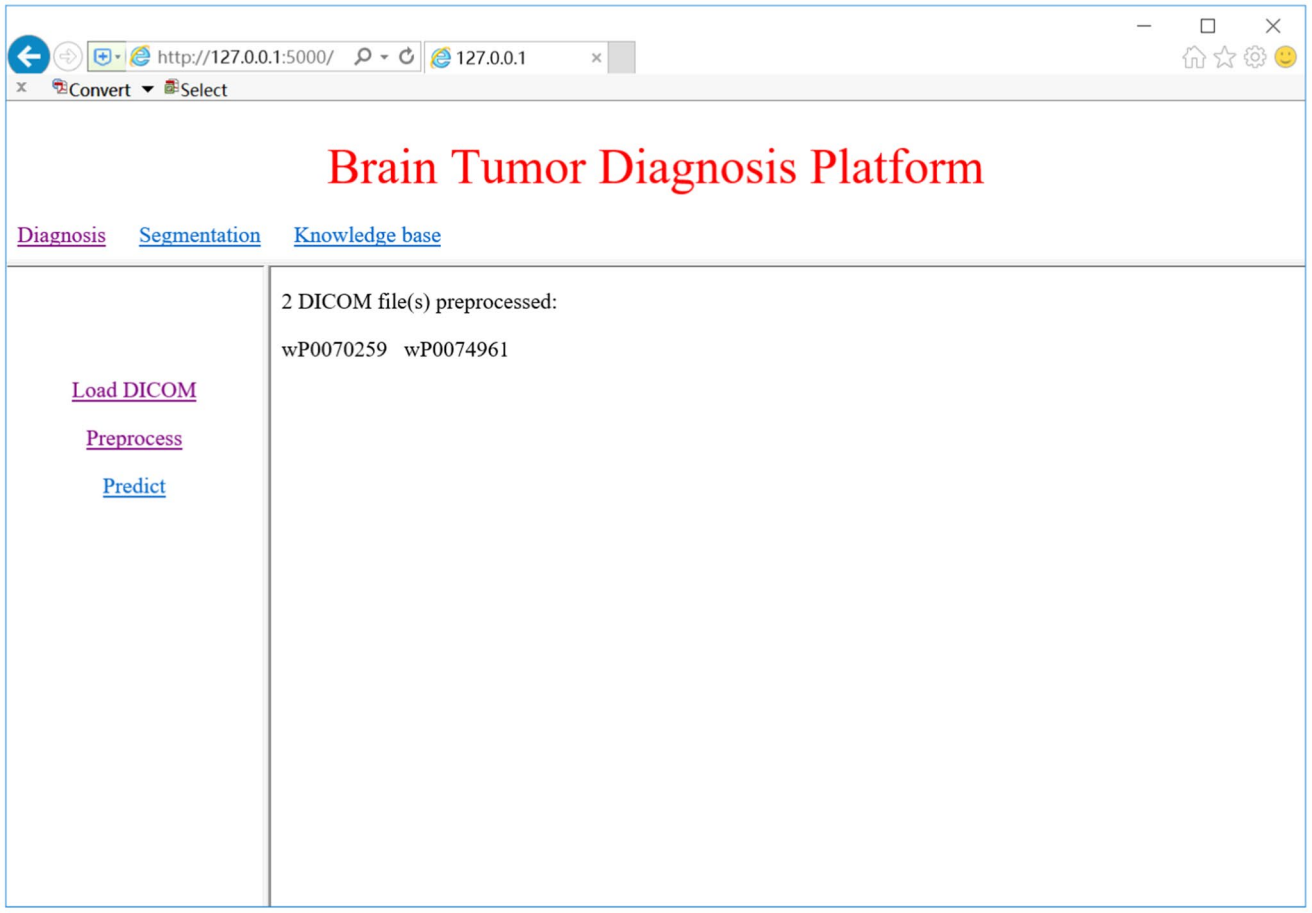
DICOM preprocessing result.

DICOM is the standard medical imaging format generated by the MRI device. When we click on the ‘Load DICOM’ link, the system prompts a dialog for choosing the folder of DICOM images. We can select the slice number to view the corresponding 2D MRI image. Once the DICOM image is loaded, we may select the ‘Preprocess’ link to begin the preprocessing process, which includes conversion from DICOM to NifTI, bias correction, Z-score normalization, and spatial normalization. Figure 9 shows the DICOM preprocessing results of the two subjects.

### Glioma diagnosis

**Figure 10:**
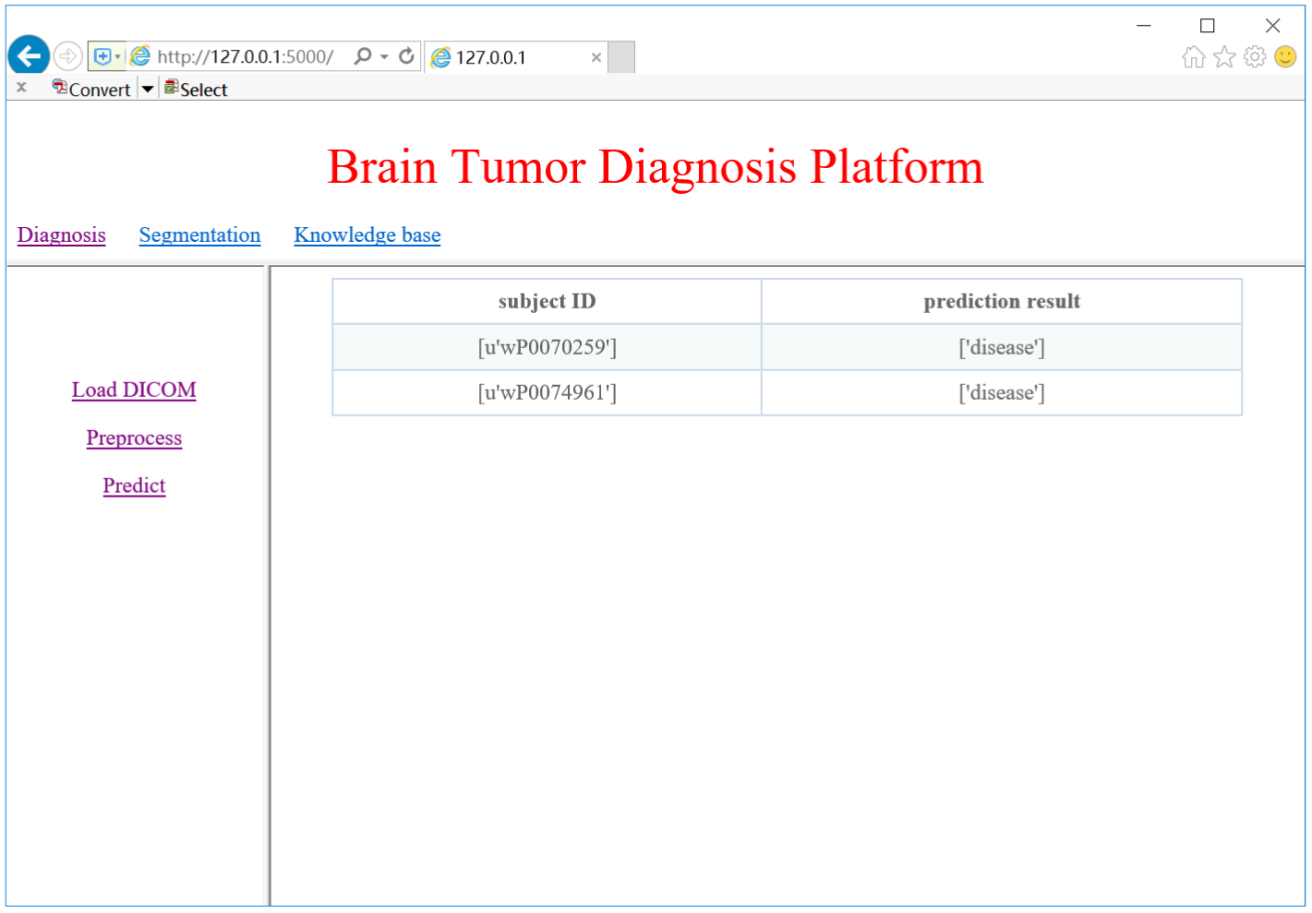
Glioma diagnosis result.

We trained two models for glioma detection and glioma grading using the DS-Detect and DS-Grading datasets, and they achieved accuracy of 86.3 and 76.3%, respectively. These models can then be used to detect gliomas and determine the grade of gliomas present in the brain. Here we take glioma detection as an example. When we click on the ‘Predict’ link, the system will make a prediction of whether the loaded DICOM images contain gliomas. Figure 10 shows the prediction results of the two subjects.

### Visualization of glioma region

**Figure 11:**
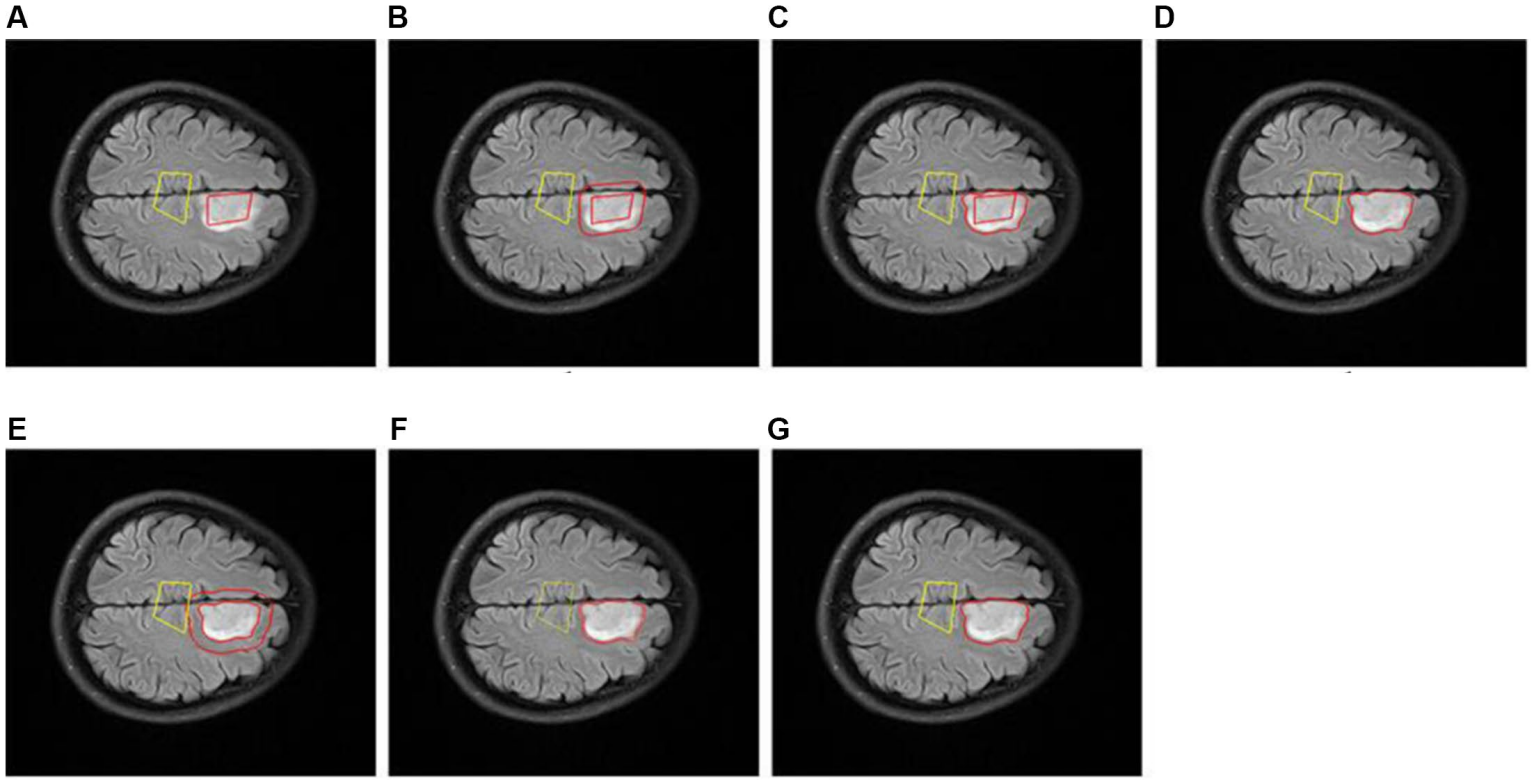
Slice glioma segmentation. **(A)** The glioma region (surrounded by red line) and normal region (surrounded by yellow line). **(B)** The expanded region (between the two red lines) in 1th iteration. **(C)** The expanded glioma region (between the two lines) in 1th iteration. **(D)** The glioma region (surrounded by red line) in 1th iteration. **(E)** The expanded region (between the two red lines) in 2th iteration. **(F)** The expanded glioma region (between the two lines) in 2th iteration. **(G)** The final glioma region (surrounded by red line).

To evaluate the segmentation effect of our proposed *Exp-seg* algorithm, we choose one patient with glioma from the DS-Detect dataset as an example, and the segmentation steps are shown in Figure 11. To obtain the best segmentation result, we choose the slice with the largest tumor area. Because *Exp-seg* is a semi-automatic segmentation algorithm, it requires the clinician to label some glioma voxels and normal voxels on the selected MRI slice (Figure 11A). Then after runs of two iterations (Figures 11B–F), the final glioma region is segmented out with red line on its contour (Figure 11G).

## Discussion

The CAD system proposed in this paper provides fundamental capabilities such as glioma classification and segmentation. For ease of use in the clinical setting, we have integrated these procedures into a web-based platform. The clinicians can operate the system *via* a web browser without the need to install extra tools like SPM, FSL, FreeSurfer etc. Another advantage of the CAD system is that we build the classification model based on machine learning techniques. Such model can be used to assign the candidate to one of the possible categories (e.g., diseased status or healthy status, high-grade or lower-grade). Different from other CAD systems based on statistical analysis, the machine learning based models enables classification or prediction on an individual level (Arbabshirani et al., 2017). And the model performance could be enhanced with expansion of the training dataset.

Glioma segmentation is performed in a very intuitive and graphical way, and the segmentation results are robust and reproducible. Although the *Exp-seg* algorithm is not fully automatic, little clinician-computer interactions are required during algorithm execution. The clinicians only need to select several voxels within the glioma region and normal region, respectively. In addition, if the segmentation result is not satisfactory, the clinicians can increase the number of voxels selected or adjust the position of selected voxels. Of course, there are still certain deficiencies in the segmentation algorithm. For example, it cannot discriminate other brain areas related to tumors such as edema and necrosis. We will try to improve the *Exp-seg* algorithm to make a more accurate segmentation in future research.

Overall, the CAD system proposed in this paper can assist the clinicians in diagnosing gliomas with machine learning models. Once the models have been trained, it can allow the clinicians to obtain the prediction results of new patients in a fast and simple way. Given the relative generality of our two-level classification framework, it is not only applicable to the diagnosis of gliomas but also to other brain conditions such as Alzheimer’s disease, Parkinson’s disease, Autism spectrum disorder, etc. Accordingly, we think that the CAD system could be a potential tool to analyze MRI images and assist in the intelligent diagnosis of brain diseases in clinical practice.

## Data availability statement

The raw data supporting the conclusions of this article will be made available by the authors, without undue reservation.

## Ethics statement

The studies involving human participants were reviewed and approved by Zhongnan Hospital of Wuhan University ethics committee. The patients/participants provided their written informed consent to participate in this study. Written informed consent was obtained from the individual(s) for the publication of any potentially identifiable images or data included in this article.

## Author contributions

QL and LL: conceptualization and supervision. TC: investigation and writing – original draft. TC and LH: methodology and software. LL and HL project administration. FX and HX resources. All authors contributed to the article and approved the submitted version.

## Funding

This research was funded by National Natural Science Foundation of China (61772375, 61936013, and 71921002), the National Social Science Fund of China (18ZDA325), National Key R&D Program of China (2019YFC0120003), Natural Science Foundation of Hubei Province of China (2019CFA025), Independent Research Project of School of Information Management of Wuhan University (413100032), the Key R&D and Promotion Projects of Henan Province of China (212102210521), and the Key Scientific Research Project in Colleges and Universities of Henan Province of China (22A520038).

## Conflict of interest

The authors declare that the research was conducted in the absence of any commercial or financial relationships that could be construed as a potential conflict of interest.

## Publisher’s note

All claims expressed in this article are solely those of the authors and do not necessarily represent those of their affiliated organizations, or those of the publisher, the editors and the reviewers. Any product that may be evaluated in this article, or claim that may be made by its manufacturer, is not guaranteed or endorsed by the publisher.

## Footnotes

^1^ http://www.cancerimagingarchive.net/
